# Foundation of the Belgian Society for Viruses of Microbes and Meeting Report of Its Inaugural Symposium

**DOI:** 10.3390/v15051213

**Published:** 2023-05-22

**Authors:** Agnieszka Latka, Abram Aertsen, Dimitri Boeckaerts, Bob Blasdel, Pieter-Jan Ceyssens, Abel Garcia-Pino, Annika Gillis, Rob Lavigne, Gipsi Lima-Mendez, Jelle Matthijnssens, Jolien Onsea, Eveline Peeters, Jean-Paul Pirnay, Damien Thiry, Dieter Vandenheuvel, Els Van Mechelen, Jolien Venneman, Gilbert Verbeken, Jeroen Wagemans, Yves Briers

**Affiliations:** 1 Laboratory of Applied Biotechnology, Department of Biotechnology, Faculty of Bioscience Engineering, Ghent University, 9000 Gent, Belgium or agnieszka.latka@uwr.edu.pl (A.L.); dimitri.boeckaerts@ugent.be (D.B.); 2 Department of Pathogen Biology and Immunology, University of Wroclaw, 51-148 Wroclaw, Poland; 3 Laboratory of Food Microbiology, Department of Microbial and Molecular Systems, Faculty of Bioscience Engineering, KU Leuven, 3001 Leuven, Belgium; abram.aertsen@kuleuven.be; 4 KERMIT, Department of Data Analysis and Mathematical Modelling, Ghent University, 9000 Ghent, Belgium; 5 Vésale Bioscience, Vésale Pharmaceutica, 5310 Noville-sur-Mehaigne, Belgium; bob.blasdel@phage.health; 6 Division of Human Bacterial Diseases, Sciensano, 1050 Brussels, Belgium; pieter-jan.ceyssens@sciensano.be; 7 Cellular and Molecular Microbiology, Faculté des Sciences, Université Libre de Bruxelles, Campus La Plaine, 1050 Brussels, Belgium; abel.garcia.pino@ulb.be; 8 Laboratory of Food and Environmental Microbiology, Earth and Life Institute, Université Catholique de Louvain, 1348 Louvain-la-Neuve, Belgium; annika.gillis@uclouvain.be; 9 Laboratory of Gene Technology, Department of Biosystems, Faculty of Bioscience Engineering, KU Leuven, 3001 Leuven, Belgium; rob.lavigne@kuleuven.be (R.L.); jeroen.wagemans@kuleuven.be (J.W.); 10 Biology of Microorganisms Research Unit (URBM), Namur Research Institute for Life Sciences (NARILIS), Université de Namur ASBL, 5000 Namur, Belgium; gipsi.limamendez@unamur.be; 11 Laboratory of Viral Metagenomics, Rega Institute, Department of Microbiology, Immunology and Transplantation, KU Leuven, 3000 Leuven, Belgium; jelle.matthijnssens@kuleuven.be; 12 Department of Trauma Surgery, University Hospitals Leuven, 3000 Leuven, Belgium; jolien.onsea@uzleuven.be; 13 Department of Development and Regeneration, KU Leuven, 3000 Leuven, Belgium; 14 Research Group of Microbiology, Department of Bioengineering Sciences, Vrije Universiteit Brussel, 1050 Brussels, Belgium; eveline.peeters@vub.be; 15 Laboratory for Molecular and Cellular Technology, Queen Astrid Military Hospital, 1120 Brussels, Belgium; jean-paul.pirnay@mil.be (J.-P.P.); gilbert.verbeken@mil.be (G.V.); 16 Veterinary Bacteriology, Department of Infectious and Parasitic Diseases, Fundamental and Applied Research for Animals and Health, Faculty of Veterinary Medicine, University of Liège, 4000 Liège, Belgium; damien.thiry@uliege.be; 17 Department of Bioscience Engineering, University of Antwerp, 2020 Antwerp, Belgium; dieter.vandenheuvel@uantwerpen.be; 18 Research Centre Health & Water Technology, University of Applied Sciences, 9000 Gent, Belgium; els.vanmechelen@hogent.be; 19 Flanders Research Institute for Agriculture, Fisheries and Food (ILVO), 9820 Merelbeke, Belgium; jolien.venneman@ilvo.vlaanderen.be

**Keywords:** Belgian Society for Viruses of Microbes, bacteriophages, triple helix model

## Abstract

The Belgian Society for Viruses of Microbes (BSVoM) was founded on 9 June 2022 to capture and enhance the collaborative spirit among the expanding community of microbial virus researchers in Belgium. The sixteen founders are affiliated to fourteen different research entities across academia, industry and government. Its inaugural symposium was held on 23 September 2022 in the Thermotechnical Institute at KU Leuven. The meeting program covered three thematic sessions launched by international keynote speakers: (1) virus–host interactions, (2) viral ecology, evolution and diversity and (3) present and future applications. During the one-day symposium, four invited keynote lectures, ten selected talks and eight student pitches were given along with 41 presented posters. The meeting hosted 155 participants from twelve countries.

## 1. Introduction

**Figure 1:**
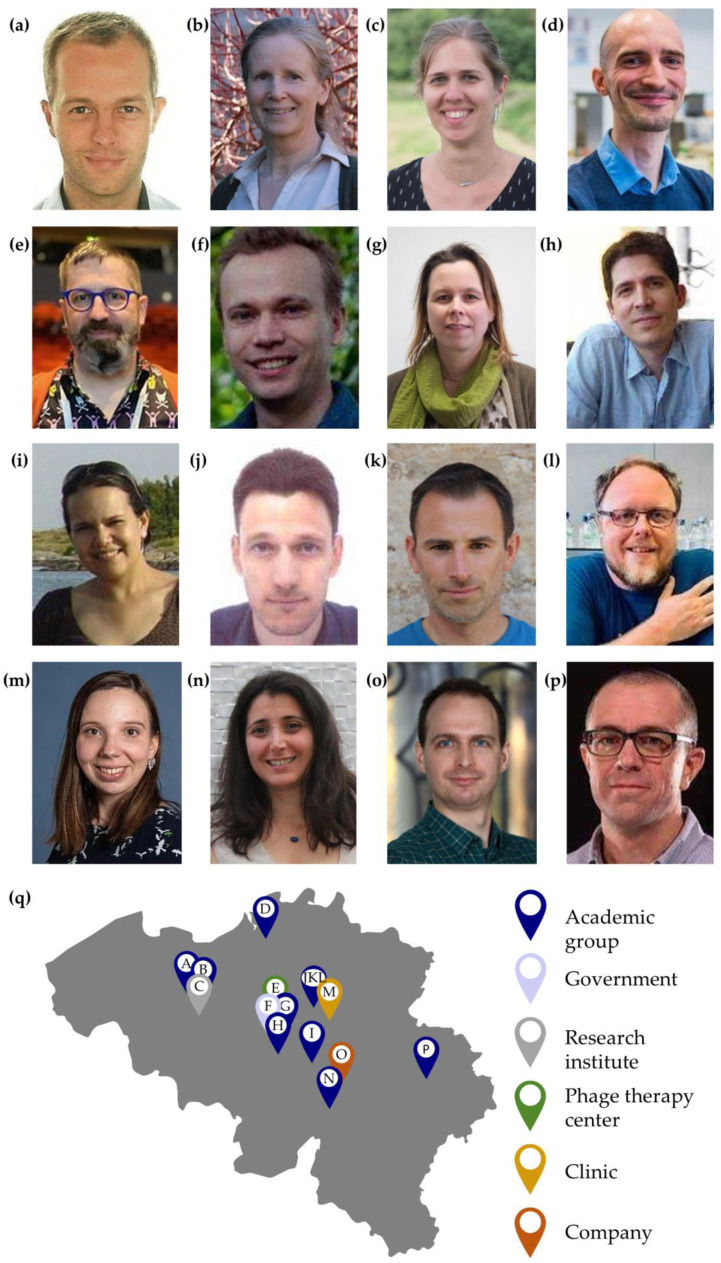
Sixteen co-founders of the Belgian Society for Viruses of Microbes and their affiliations. From west to east: (**a**) Ghent University (Yves Briers); (**b**) University of Applied Sciences Ghent (Els Van Mechelen); (**c**) Flanders Research Institute for Agriculture, Fisheries and Food (ILVO) (Jolien Venneman); (**d**) University of Antwerp (Dieter Vandenheuvel); (**e**) Queen Astrid Military Hospital (Jean-Paul Pirnay); (**f**) Sciensano–Federal agency of public health (Pieter-Jan Ceyssens); (**g**) Vrije Universiteit Brussel (Eveline Peeters); (**h**) Université libre de Bruxelles (Abel Garcia-Pino); (**i**) UCLouvain (Annika Gillis); (**j**) KU Leuven (Abram Aertsen), (**k**) KU Leuven (Jelle Matthijnssens), (**l**) KU Leuven (Rob Lavigne); (**m**) University Hospitals Leuven (Jolien Onsea); (**n**) University of Namur (Gipsi Lima-Mendez); (**o**) Vésale Bioscience (Bob Blasdel); (**p**) University of Liège (Damien Thiry); (**q**) Geographical location of the different founders. The labels correspond to the different founders from the previous panels.

Belgium holds a long-standing position in the field of microbial viruses, dating back to the early works of André Gratia on *Staphylococcus aureus* phages and the first experimental therapeutic use of phages by Richard Bruynoghe and their student Joséph Maisin, both in 1921 [1]. René Appelmans developed a liquid method of phage titration called the “Appelmans protocol”, which became the base for the preparation of a large number of phage cocktails targeting a broad range of pathogenic bacteria in Georgia [2,3] and is nowadays frequently used for phage adaptation to bacterial hosts, which is known as “phage training”. Pioneering work in the field continued with the discovery of positive regulation of gene expression in phage lambda by René Thomas and coworkers in 1966, the first viral genome (MS2) sequenced by the group of Walter Fiers in 1976 and, more recently, a highly advanced regulatory framework for phage therapy actively applied in Belgian hospitals, known as magistral phage therapy [4,5,6]. Today, Belgium has a vibrant and diverse community of phage (and other microbial virus) researchers. Based on this premise, the Belgian Society for Viruses of Microbes (BSVoM) was founded as a non-profit organization (www.bsvom.be, accessed on 20 May 2023) on 9 June 2022, with its associated articles being published in the Belgian official gazette. The BSVoM aims to sustain and improve the dense Belgian research and development network on viruses of microbes by bringing together all stakeholders from academia, government and industry. Moreover, this national society is formally associated with the International Society for Viruses of Microorganisms (www.isvm.org, accessed on 20 May 2023). In spite of the small geographical size of Belgium, at least fourteen entities are currently active in the field of viruses of microbes, all represented by at least one co-founder (Figure 1). In its foundation assembly, Prof. Annika Gillis (UCLouvain), Dr. Pieter-Jan Ceyssens (Sciensano) and Prof. Yves Briers (Ghent University) were elected as secretary, treasurer and chair for three years. Our society adopts the triple helix model, providing an interdisciplinary perspective ranging from basic research to industrial developments and biotechnological and clinical applications. The BSVoM pursues the intensification of this fertile Belgian ecosystem on viruses of microbes, with a particular focus on supporting young and future generations in the field. The society counts today 237 members (status on 11 May 2023) and is supported by Vésale Bioscience (phage.health, accessed on 20 May 2023) as its main society sponsor. Vésale Bioscience is a Belgian biotech company created in 2018 as a spin-out of Vésale Pharma, developing personalized phage therapy solutions.

**Figure 2:**
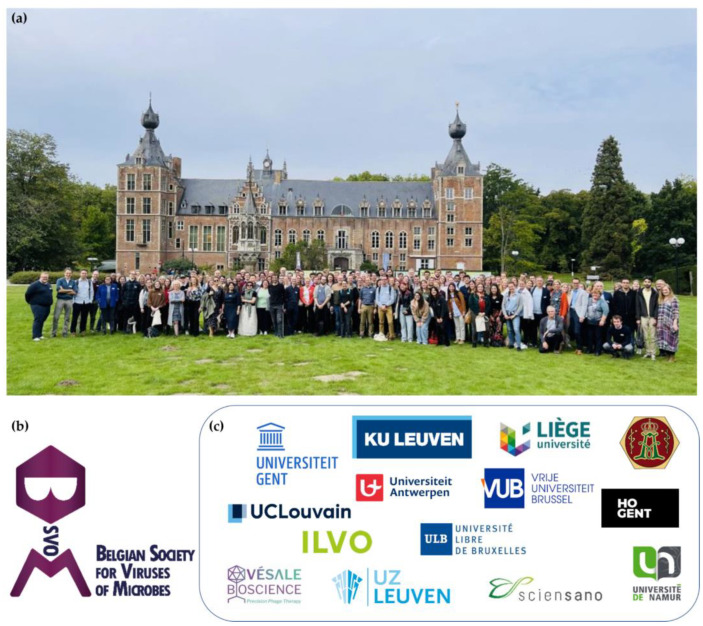
(**a**) Group picture of participants of the inaugural BSVoM symposium in front of Arenberg Castle (Leuven, Belgium) (155 participants from 12 different countries). (**b**) BSVoM logo. (**c**) Affiliations of BSVoM founders.

**Figure 3:**
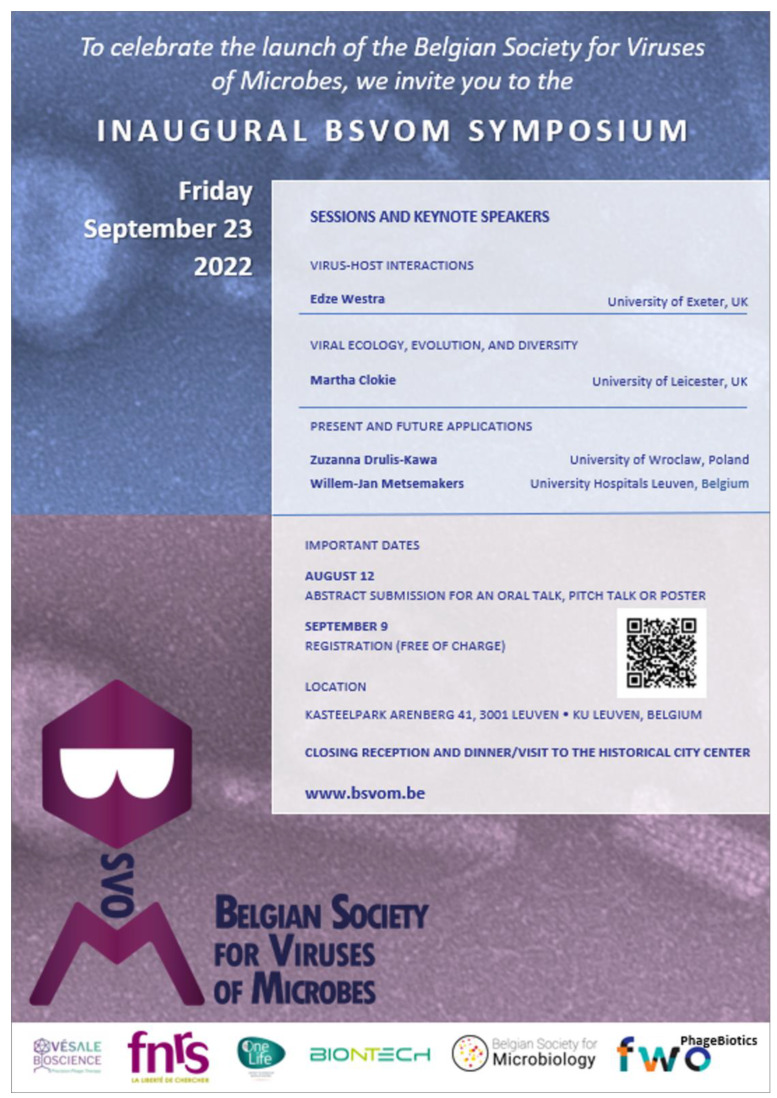
Flyer advertising the inaugural BSVoM symposium.

The inaugural BSVoM symposium took place on 23 September 2022 at the Thermotechnical Institute in Leuven, Belgium, with 155 participants from 12 countries (Figure 2). The symposium program combined three thematic sessions covering fundamental and translational aspects of research on viruses of microbes: (1) virus–host interactions, (2) viral ecology, evolution and diversity and (3) present and future applications (Figure 3). The sessions were launched by keynote speakers Prof. Edze Westra (University of Exeter, UK), Prof. Martha Clokie (University of Leicester, UK), Prof. Zuzana Drulis-Kawa (University of Wroclaw, Poland, current president of the International Society for Viruses of Microorganisms) and Prof. Dr. Willem-Jan Metsemakers (UZ Leuven). In addition, ten talks were selected from the submitted abstracts and a pitch session with eight pitches from young upcoming talents was organized prior to the poster session with 41 posters being presented (Table 1). There were ample opportunities to discuss the most recent advances, including a social activity to foster interactions. 

## 2. Scientific Sessions

The first session on virus–host interactions, chaired by Prof. Abel García-Pino (Université libre de Bruxelles, Brussels, Belgium), began with a keynote lecture by Prof. Edze Westra of the University of Exeter, Exeter, UK, who discussed their lab’s research into the ecology and evolution of bacterial defense systems against phage predation. Westra has studied both the molecular and evolutionary aspects of bacteria–phage interactions, focusing on the CRISPR-Cas adaptive immune system acting as a genetic memory to detect and destroy re-infecting phages that carry a cognate target sequence. Westra’s lab combines genomics, mathematical modelling and experimental analyses of *Pseudomonas aeruginosa* to determine when different phage defense mechanisms are favored over others and how they shape the evolutionary epidemiology of the phage and virulence evolution of the pathogen. Westra’s group has received several prestigious awards for its work.

The next speaker was Kaat Schroven of KU Leuven (Leuven, Belgium), who presented research on lytic phages as a treasure trove for virulence attenuating proteins against *P. aeruginosa*. Schroven’s research involves performing several high-throughput analyses using a library of individually expressed proteins from lytic phages to identify potential effectors targeting virulence factors of *P. aeruginosa*. Schroven discovered three different phage ORFans that specifically and significantly attenuate key virulence factors of the pathogen, including the type IV pili, the type 2 secretion system and ExoS (a type 3 secretion system product). The identified phage-encoded virulence attenuators expand the diversity of regulatory mechanisms encoded by phages to impact bacterial physiology and may serve as a source for diverse biotechnological applications [7,8].

The topic of the first session switched towards Gram-positive host cells with the talk of Dr. Lionel Schiavolin, affiliated with Université Libre de Bruxelles. They discussed the tripartite interactions of Group A *Streptococcus* (GAS), specific phages and the human host. Utilizing RNA-Seq, they deciphered phage–GAS interactions and found a reprogramming of up to 50% of the transcriptome of an M25 strain during infection by the virulent phage A25. The most downregulated genes belonged to the fatty acid synthesis (FASII) pathway. They also found that serum protects GAS from phage infection, while the addition of human serum albumin and fatty acids increases bacterial clearance. 

An important topic from the phage therapy point of view was covered by Prof. Stan Brouns (Delft University of Technology, Delft, The Netherlands). With their lecture entitled “Accumulation of defense systems drive panphage resistance in *P. aeruginosa*”, they discussed the problem of clinical strains of *P. aeruginosa*, which are equipped with multiple phage defense systems. Their research group demonstrated that the major determining factor for the host spectrum of phages specific to clinical strains is the intracellular defense system. Multiple systems can provide complementary and overlapping phage protection, resulting in panphage resistance. They also discovered that mobile genetic elements with phage defense systems can be exchanged via horizontal transfer, resulting in up to 19 phage defense systems per single *P. aeruginosa* clinical isolate. The data are available as a preprint under the same title [9].

Phages specific to another ESKAPE pathogen, *Klebsiella pneumoniae*, were the subject of collaborative research between Ghent University (Belgium) and the University of Wroclaw (Poland). Dr. Agnieszka Latka’s presentation focused on phage KP32 receptor-binding proteins with depolymerizing activities. A crystal structure analysis revealed a multimodular structure with a central enzymatic domain, two additional C-terminal domains, a carbohydrate-binding module (CBM) and a lectin-like domain (LD) [10]. C-terminally truncated proteins, chimeric protein fusions and phage particles lacking the domains of interest were prepared to analyze the function of the domains. It was concluded that the lectin-like domain is essential for phage infection. No serotype specificity switch was observed after chimeric fusions of C-terminal domains with other receptor binding proteins (RBPs). The CBM and LD were not able to bind to the surface of capsulated bacteria. Further research is needed to elucidate the exact function of the CBM and LD. 

The second session of the conference, chaired by Prof. Annika Gillis, focused on viral ecology, evolution and diversity. The keynote speaker was Prof. Martha Clokie, a renowned expert in bacteriophage biology from the University of Leicester in the United Kingdom, who is devoted to developing phages as therapeutics for humans and animals. Their work uses genomic and structural approaches to identify traits associated with phage efficacy and to study phage–bacterial interactions in relevant models. Martha Clokie has pioneered research into *Clostridium difficile* phages, an important pathogen causing infectious diarrhea, with the view to developing them for next generation therapeutics. Their work has included unraveling phage diversity using computational, structural and phenotypic approaches and understanding the resistance rates towards phages compared to antibiotics. Furthermore, they have optimized ex situ models to study *C. difficile*–phage interactions in epithelial cells and optimized biofilm, *Galleria mellonella* and artificial gut models to better understand application regimens, dosages and efficacies [11,12,13]. Additionally, Martha Clokie has developed and regularly runs a course to teach phage biology to African academics (as part of Gates-funded “Phages for Global Health”). 

Next, Daan Jansen from KU Leuven presented a study to use community-typing as a tool to explore virome compositional changes in inflammatory bowel disease (IBD) patients (a group of chronic inflammatory diseases of the gut) [14]. Viral metagenomics and deep sequencing of fecal samples from 181 patients undergoing immunomodulatory therapy were performed, stratifying them into two viral community types. Variations in the gut virome were explained by factors such as patients’ individuality, disease location, age and fecal moisture. The endoscopic outcome was associated with gut virome variations. The findings suggest that viral community typing could be used to gain a deeper understanding of IBD subtypes or as a potential biomarker in the future.

The second scientific session was concluded with a presentation by Prof. Evelien Adriaenssens from the Quadram Institute Bioscience in the United Kingdom, who discussed the importance of phages in the healthy human gut microbiome. The advances in sequencing technology and bioinformatics have enabled the reconstruction of complete phage genomes, unlocking the investigation of phage diversity [15]. Evelien Adriaenssens presented studies of the diversity and activity of phages in two cohorts of healthy individuals, PEARL (mothers and infants) and MOTION (older population). Using these results, they seek to explore how phages can be used to improve health across life.

A pitch session chaired by Dr. Pieter-Jan Ceyssens was organized in the middle of the symposium and preceding the poster session with the particular goal to give a first-stage experience to junior researchers and to attract attention to their poster (Table 1). In total, eight pitches were selected. Lore Van Espen (KU Leuven) spoke about the increased human gut phage diversity found in patients with acute-on-chronic liver failure. Rémy Dugauquier (Université de Namur, Université libre de Bruxelles, Belgium) shared the development of ADAM, an automatic detection of new antiviral mechanisms. Niels Vander Elst (KU Leuven, Ghent University) discussed a method for the purification of non-pyrogenic endolysins with an in vitro or in vivo application. Hisham Shaikh (Flanders Marine Institute (Belgium), Ghent University, Utrecht University, the Netherlands) talked about the diversity and ecology of marine viruses in the coastal and open North Sea. Manon Nuytten (Université Catholique de Louvain) presented the characterization of holins encoded by two phages to exit their *Bacillus cereus* host. Vincent De Maesschalck (KU Leuven, Ghent University) discussed the development of a bioluminescent ex vivo wound model to characterize novel phage-inspired enzybiotics [16]. Sam van Beljouw (Delft University of Technology, Kavli Institute of Nanoscience in the Netherlands) presented their research on Craspase, a self-regulating protease that is activated in a sequence-specific manner by viral RNA. In addition, Céline Antoine (Université de Liège, Belgium) presented their research on the in vitro characterization and in vivo assessment of newly isolated phages against *Escherichia coli* K1 [17]. The best pitch talk award went to Sam van Beljouw.

The third session, focusing on the present and future applications of phages, was chaired by Dr. Jean-Paul Pirnay from the Queen Astrid Military Hospital (Belgium). In this session, two keynote lectures were presented: the first by Prof. Zuzanna Drulis-Kawa (University of Wroclaw, Wrocław, Poland), an executive committee member of the ESCMID Study Group for Nontraditional Antibacterial Therapy–ESGNTA and current president of the International Society for Viruses of Microbes, and the second by Prof. Dr. Willem-Jan Metsemakers, a trauma surgeon at the Department of Trauma Surgery of University Hospitals Leuven (UZ Leuven, Belgium) and associate professor at KU Leuven. Prof. Zuzanna Drulis-Kawa’s research focus is the antibacterial activity of phages and phage-derived depolymerases and endolysins specific to ESKAPE pathogens (*K. pneumoniae* and *P. aeruginosa*). In her keynote lecture, she discussed the role of phage-borne depolymerases in bacteriophage–bacteria–host interactions. Bacterial surface glycans serve as molecular patterns, recognized by the immune system. Via glycan digestion or modification, bacteria can become sensitive to host immune responses. Prof. Zuzanna Drulis-Kawa demonstrated how depolymerases can be exploited for new diagnostics and therapeutic approaches against infectious diseases. She emphasized the need to deepen the knowledge of the phage enzyme structure and domain build-up, as well as in vitro and in vivo enzyme specificity and stability. Prof. Dr. Willem-Jan Metsemakers talked about bacterial infections as a major complication in orthopedics. Their incidence continues to rise due to higher numbers of both elective joint replacements and operatively treated fractures. Biofilms on the surface of implanted devices, intracellular bacteria and bacteria within canaliculi are known to cause infections. To address this, antibiotic therapy has been utilized; however, this is associated with side effects and the development of antimicrobial resistance. Investigations into the use of phage therapy for musculoskeletal infections are sparse; most of the knowledge coming from the Eliava Institute of Bacteriophages, Microbiology and Virology (Tbilisi, Georgia). Metsemakers is involved in multiple research projects on the topic of implant-related infections and reported a rising number of studies/instances in recent years [18,19,20]. They are the head of the care program for musculoskeletal infections and of the Coordination Group for Bacteriophage Therapy, Leuven, affiliated with Leuven University Hospitals. They stressed that bacteriophage therapy is a useful adjunct for the treatment of difficult-to-treat infections; however, optimization of application techniques and further research is still needed.

Johan Quintens from Vésale Biosciences (Belgium) discussed the triple helix model of collaboration between industry, academics and government. They explained that the three players have synergistic functions: universities engaging in basic research, companies producing commercial goods and governments regulating markets. This model stimulates innovation by valuing the role of universities as creators of knowledge and enabling entrepreneurial activity by capitalizing on knowledge. Knowledge transfer between universities and industry is a critical aspect, for example, through technology transfer offices and joint projects. Governments act as a facilitator, providing funding and policy-making tools for economic growth and regional development. This form of innovation is most successful in a knowledge-based society. In the context of phage therapy, Vésale Biosciences has collaborated with LabMCT (Queen Astrid Military Hospital, Dr. Jean-Paul Pirnay), signing a Triple Helix agreement in 2020 with the main goals of commercializing the personalized phage therapy model, developing a value chain from diagnostic to production and distribution and working closely with the academic and government sectors.

Plant-related phage application was discussed by Dr. Gil Luypaert and Anneleen Volckaert (De Ceuster Meststoffen nv (DCM), Belgium). They presented that DCM is developing a phage-based plant protection product to control fire blight caused by *Erwinia amylovora*. Field trials at different locations within the EU have yielded promising efficacy data, with more than 90% efficacy being reported under natural infection pressure in open field conditions, while studies under greenhouse conditions with artificial high-dose *E. amylovora* inoculation were more challenging, requiring an appropriate multiplicity of infection (MOI). However, current EU plant protection regulations pose a challenge to registration due to the narrow host range of bacteriophages. A flexible regulatory framework that allows variability in the composition of a bacteriophage cocktail is needed to ensure its efficacy against any isolate of the bacterial target. This could provide a sustainable and biological alternative to chemical control of diseases.

The final talk of the conference was given by Steven De Soir from UCLouvain and Queen Astrid Military Hospital on the potential of a novel treatment modality for bacterial-biofilm-related infections on orthopedic implants. They studied the synergy between de novo isolated phages and routinely used antibiotics, such as ciprofloxacin, meropenem and ceftazidime. Significant reductions in CFU counts and biofilm biomass were seen when applying antibiotics in combination with bacteriophages. Scanning electron microscopy of the *Pseudomonas aeruginosa* PAO1 biofilms, grown on titanium coupons and treated with phages in combination with ciprofloxacin, confirmed these findings. It was concluded that a combination of phages and antibiotics is more efficient than either type of agent alone. Furthermore, the sequence of therapy (phage treatment first or antibiotic treatment first) resulted in the same reduction in infecting cells; however, a simultaneous combination therapy of both phages and antibiotics further diminished the number of pathogens present. Further research on optimizing the conditions of exposure is ongoing.

## 3. Conclusions

The BSVoM was founded to bring together the Belgian microbial virus community active in academia, industry, clinics and government. The enthusiasm present at its inaugural symposium was encouraging and underlines the benefits and opportunities of this initiative. A community that wishes to grow invests in its junior researchers. This goal was addressed by the pitch session, the numerous posters and follow-up initiatives such as a phage hands-on workshop in the Queen Astrid Military Hospital under the supervision of Dr. Jean-Paul Pirnay, Dr. Tea Glonti and Dr. Maia Merabishvilli. In addition, the “My hero and me” webinar series was launched, giving junior researchers the opportunity to present a back-to-back webinar with their scientific heroes in the phage field that excel in their domain. The society further capitalized on its strong triple helix interactions already pre-existent to the foundation. Bringing all stakeholders together in a single community and symposium stimulates fruitful interactions and new initiatives. We are looking forward to the second edition of the BSVoM symposium, which will be held on 8 September 2023 at the University of Liège, Belgium.

## Figures and Tables

**Table 1:** Posters presented during the inaugural BSVoM symposium. Best poster award winners are underlined. Authors indicated by asterisks contributed equally. ‡ Deceased.

Authors	Poster Title
Antoine Céline, Laforêt Fanny, Blasdel Bob, Fall Abdoulaye, Duprez Jean-Noël, Mainil Jacques, Delcenserie Véronique, Thiry Damien	In vitro characterization and in vivo assessment of newly isolated phages against *Escherichia coli* K1
Bäcker Leonard *, Staes Ines *, Simoens Kenneth, De Winter Kjerstin, Marolt Gasper, Cenens William, Wolput Sanne, Vazquez Alan, Goos Peter, Lavigne Rob, Bernaerts Kristel, Aertsen Abram	Phage superinfection exclusion factors against host cell scarcity
Boeckaerts Dimitri, Stock Michiel, De Baets Bernard, Domingo-Calap Pilar, Sanjuan Rafael, Briers Yves	Lessons learned from predicting the structures of over 100 *Klebsiella* phage receptor-binding proteins
Boon Maarten, De Zitter Elke, Putzeys Leena, Kuznedelov Konstantin, Severinov Konstantin, Van Meervelt Luc, Lavigne Rob	‘Drc’ matters: A small ORFan with a big role in phage transcription regulation
Campobasso Claudia, Wagemans Jeroen, Lavigne Rob, Tavanti Arianna, Di Luca Mariagrazia	Isolation and characterization of the novel *Staphylococcus aureus* bacteriophage φZeno
Cepauskas Albinas, Tamman Hedvig, Zhang Tong, Coppieters Wallant Kyo, Kurata Tatsuaki, Talavera Ariel, Marteens Chloe, Hauryliuk Vasili *, Garcia-Pino Abel *, Laub Michael *	Direct activation of an innate immune system in bacteria by a viral capsid protein
Landlinger Christine, Oberbauer Vera, Podpera Tisakova Lenka, Schwebs Timo, Berdaguer Rocío, van Simaey Leen, Vaneechoutte Mario, Corsini Lorenzo	Preclinical data on the *Gardnerella*-specific endolysin PM-477 indicate its potential to improve the treatment of bacterial vaginosis through enhanced biofilm removal and avoidance of resistance
Cremelie Emma, De Groote Philippe, Lamote Babette, Briers Yves	Hacking yeast cells with phage lytic proteins to win the fight against antibiotic resistant bacteria
Criel Bjorn, Van Criekinge Wim, Briers Yves	Exploring PhaLP, an open-source portal for Phage Lytic Proteins, to engineer modular enzybiotics against vancomycin-resistant *Enterococcus faecalis*
Dams Dorien, Latka Agnieszka, Hulsens Mathilde, Drulis-Kawa Zuzanna, Briers Yves	Development of a synthetic, VersaTile strategy for receptor-binding protein engineering of *Klebsiella pneumoniae* phages
De Jode Mathieu, Willocx Marie, Djebara Sarah, Pirnay Jean-Paul, Ceyssens Pieter-Jan	Current and future clinical phage product quality control in Belgium
De Maesschalck Vincent, Briers Yves, Vande Velde Greetje, Lavigne Rob	Development of a bioluminescent ex vivo wound model to characterize novel phage-inspired enzybiotics
Desmecht Salomé, Antoine Céline, Laforêt Fanny, Duprez Jean-Noël, Schonbrodt Alain, Lieffrig François, Thiry Damien	Isolation and characterization of four new bacteriophages against *Aeromonas salmonicida*, the causative agent of furunculosis
De Smet Jeroen	Unraveling the gut virome in Black Soldier Fly larvae (*Hermetia illucens*)
Dugauquier Rémy, Ainelo Andres, Pelzer Lara, Garcia-Pino Abel, Lima-Mendez Gipsi	ADAM: Automatic Detection of new Antiviral Mechanisms
Duyvejonck Lisa, Gerstmans Hans, Stock Michiel, Grimon Dennis, Lavigne Rob, Briers Yves	Diverse configurations of VersaTile-engineered lysins: A rapid and high-throughput evaluation
Fortuna Kiandro, Holtappels Dominique, Rodrigues Savio, Verwilt Poi, Rediers Hans, Baeyen Steve, Van Vaerenbergh Johan, De Coninck Barbara, Maes Martine6, Lavigne Rob, Wagemans Jeroen	Phage biocontrol of rhizogenic *Agrobacterium*: Solving the root of the problem
Goossens Michael, Glonti Téa, Dams Dorien, Łątka Agnieszka, Pirnay Jean-Paul, Briers Yves	SynPhage: The construction of a synthetic *Escherichia* virus T7
Habets Audrey, Antoine Céline, Wagemans Jeroen, Vermeersch Marjorie, Laforêt Fanny, Lavigne Rob, Mainil Jacques, Thiry Damien	Phage-mediated Shiga-toxin gene transduction from O80:H2 Shiga toxigenic *E. coli* (STEC) to non-STEC strains and in vivo virulence assessment
Hendrix Hanne, Itterbeek Annabel, Longin Hannelore, Vriens Eveline, Vallino Marta, van Noort Vera, Boon Maarten, Lavigne Rob	A novel regulator of type IV pili assembly, PlzR, induces phage resistance in *Pseudomonas aeruginosa*
Itterbeek Annabel, Colak Yunus, Lavigne Rob, Paeshuyse Jan	Nanobodies as a new diagnostic tool for *Mycobacterium bovis*—optimization of alpaca nanobody displaying phage library preparation
Laforêt Fanny, Antoine Céline, Blasdel Reuter Bob, Detilleux Johann, Pirnay Jean-Paul, Brisse Sylvain, Fall Abdoulaye, Duprez Jean-Noël, Delcenserie Véronique *, Thiry Damien *	In vitro and in vivo assessments of two bacteriophages against a urinary tract infection *Klebsiella pneumoniae*
Lammens Eveline-Marie, Boon Maarten, Grimon Dennis, Briers Yves, Lavigne Rob	SEVAtile: A standardized DNA assembly method optimized for *Pseudomonas*
Lechuga Ana, Lood Cédric, Berjón Mónica, del Prado Alicia, Wagemans Jeroen, van Noort Vera, Lavigne Rob, Salas Margarita ‡, Redrejo-Rodríguez Modesto	A yeast two hybrid-high throughput sequencing approach for unraveling interactions between Bam35 and its *Bacillus* host
Longin Hannelore, Hendrix Hanne, Lavigne Rob, van Noort Vera	Structural exploration of the impact of phage-driven lysine acetylations on methionine synthase dynamics
Molendijk Michèle, Bode Lonneke, Phan My, Strepis Nikolas, Worp Nathalie, Nieuwenhuijse David, Schapendonk Claudia, Verbon Annelies, Koopmans Marion, de Graaf Miranda, van Wamel Willem	The efficacy of bacteriophages against clinical *Staphylococcus aureus* strains under physiological relevant conditions
Nuytten Manon, Leprince Audrey, Gillis Annika, July Elise, Tesseur Coralie, Mahillon Jacques	Getting outside the cell: Characterization of holins encoded by two phages to exit their *Bacillus cereus* host
Olejniczak Sebastian, Otwinowska Aleksandra, Maciejewska Barbara, Łątka Agnieszka, Squeglia Flavia, Briers Yves, Berisio Rita, Drulis-Kawa Zuzanna	Insight into key structural points of capsule-targeting phage depolymerases specific to *Klebsiella pneumoniae* K63
Pas Célia, Dams Dorien, Fieseler Lars, Briers Yves	Engineered phage-tail-like bacteriocins targeting STEC pathogens
Poppeliers Jorien, Putzeys Leena, Boon Maarten, Lavigne Rob	Bacteriophage LUZ100: an unusual temperate member of the Autographiviridae family infecting *Pseudomonas aeruginosa*
Pottie Iris, Duyvejonck Lisa, Briers Yves	Phages in feces: Developing a platform for discovery of *Enterococcus* lysins
Putzeys Leena, Boon Maarten, Poppeliers Jorien, Lammens Eveline-Marie, Kuznedelov Konstantin, Severinov Konstantin, Lavigne Rob	ONT-capable-seq: A new approach to explore the transcriptional architecture of bacterial viruses
Shaikh Hisham Mohammed, Brussaard Corina Paula Dorothea, De Rijcke Maarten	Diversity and ecology of marine viruses in the coastal and open North Sea
Steinmetz Jenny, Schiavolin Lionel, Smeesters Pierre, Botteaux Anne	Analysis of Group A *Streptococcus* lytic phage interactions with human serum
van Beljouw Sam, Hu Chunyi, Haagsma Anna, Rodríguez-Molina Alicia, van den Berg Daan, Vink Jochem, Ke Ailong, Brouns Stan	Craspase is a self-regulating protease that is sequence specifically activated by viral RNA
van den Berg Daan, van der Steen Baltus, Costa Ana Rita, Brouns Stan	Phage tRNAs evade tRNA-targeting host defenses through anticodon loop mutations
Vander Elst Niels, De Maesschalck Vincent, De Groote Philippe, Meyer Evelyne, Lavigne Rob *, Briers Yves *	A method for the purification of non-pyrogenic endolysins with an in vitro or in vivo application
Vander Elst Niels, Linden Sara, Meyer Evelyne, Lavigne Rob, Briers Yves, Nelson Daniel	Characterization of endolysins PlySs-2 and -9 with in vitro lytic activity against bovine mastitis *Streptococcus uberis*
Van Espen Lore, Close Lila, Papp Maria, Trebicka Jonel, Matthijnssens Jelle, MICROBPREDICT partners	Human gut phage diversity is increased in patients with acute-on-chronic liver failure
Wicke Laura, Gerovac Milan, Vogel Jörg, Lavigne Rob	Hunting for phage-encoded small non-coding RNA transcripts
Wittouck Stijn, van Sinderen Douwe, Mahony Jennifer, Lebeer Sarah	The phageome of *Lactobacillaceae*

## Data Availability

Data sharing not applicable. No new data were created or analyzed in this study. Data sharing is not applicable to this article.
